# *“I feel more comfortable speaking to a male”*: Aboriginal and Torres Strait Islander men’s discourse on utilizing primary health care services

**DOI:** 10.1186/s12939-018-0902-1

**Published:** 2018-12-17

**Authors:** Kootsy Canuto, Gary Wittert, Stephen Harfield, Alex Brown

**Affiliations:** 1 Wardliparingga Aboriginal Research Unit, South Australian Health and Medical Research Institute, Adelaide, Australia; 2 Freemasons Foundation Centre for Men’s Health, University of Adelaide, Adelaide, Australia; 3 Centre of Research Excellence in Aboriginal Chronic Disease Knowledge Translation and Exchange (CREATE), University of Adelaide, Adelaide, Australia; 4 School of Public Health, the University of Adelaide, Adelaide, Australia; 5 Sansom Institute for Health Research, University of South Australia, Adelaide, Australia

**Keywords:** Aboriginal, Torres Strait Islander, Indigenous, Men’s health, Primary health care services, Health service utilization

## Abstract

**Background:**

Aboriginal and Torres Strait Islander men have the highest morbidity and mortality rates, and lowest rates of health service utilization in Australia. There is a current perception that Aboriginal and Torres Strait Islander men are disinterested in their health. This study aimed to identify the perceived motivators, barriers and enablers of Aboriginal and Torres Strait Islander men’s utilization of primary health care services, explore their experiences and obtain suggestions from them as to how services could be modified to improve utilization.

**Methods:**

This study utilized the principles of Indigenist Research Methods. Semi-structured interviews with Aboriginal and Torres Strait Islander men (*N* = 19) took place in South Australia and far north Queensland. Participants were asked about their experiences with primary health care services, including what they could remember as a child. A thematic analysis of the qualitative data was completed without the use of computer software.

**Results:**

Feelings of invincibility, shame, being uncomfortable, fearful, along with long waiting times, having a lack of knowledge, and culturally inappropriate staff/services were all found to be barriers to service utilization. Enabling factors included convenience, the perceived quality of the service, feeling culturally safe and/or a sense of belonging, and having a rapport with staff. Motivation for attending primary health care services included going when feeling sick/unwell, attending a particular service (dental or sexual health), visiting for check-ups and preventative health and family encouragement.

This study also highlights strategies surrounding logistical factors, promotion of services and improved communications, having culturally appropriate services and providing gender specific services all of which were suggested by the participants to improve service utilization.

**Conclusion:**

Contrary to common misperceptions, this study demonstrated that most of the Aboriginal and Torres Strait Islander men participants were motivated to engage with primary health care services for preventative health care. Even though there were men that fitted the stereo-type who avoid doctors, there were usually underlying reasons and barriers accounting for this reluctance. This study suggests that if primary health care services commit to better understanding the barriers, enablers and motivators their cohort of men face, then utilization could be greatly improved.

## Background

The lives of Aboriginal and Torres Strait Islander men are shaped by unique historical, socio-cultural economic, environmental and political factors that impact on their psychological and physical wellbeing. Available data suggests that they are worse off than any other population group in Australia. Aboriginal and Torres Strait Islander men are at higher risk of committing suicide and self-harm, engaging with the justice system, developing drug and alcohol related illnesses, being afflicted by psychological illness [2, 6, 7] and premature and severe cardiovascular disease, type 2 diabetes and associated complications [6]. The extent to which inadequate utilization of health services contributes to the problems experienced is unclear, as comprehensive data relating to Aboriginal and Torres Strait Islander health service use remains limited [9]. What is clear is that Aboriginal and Torres Strait Islander men are the lowest users of primary health care services (PHCSs) and tend to delay care, often presenting when their situation has significantly progressed, and their illness is serious [2, 5, 10].

There is acknowledgment that an increased focus on men’s health should occur [17, 19]. However, there is little research that has addressed the needs of Aboriginal and Torres Strait Islander in terms of access to, and outcomes from, PHCSs. Such data are required to inform improvements in policy and practice.

This study aimed to (1) identify the perceived motivators, barriers and enablers of Aboriginal and Torres Strait Islander men’s utilization of PHCSs, (2) explore the experience of these men in doing so and (3) obtain suggestions as to how services could be modified to improve utilization.

## Methods

### Study design

To better understand the motivators, barriers and enablers of Aboriginal and Torres Strait Islander men’s utilization of PHCSs it was important to document their narratives. This research uses an ethnographic methodology grounded in an Indigenist research approach, which places value on Indigenous knowledge and privileges Indigenous voices and Indigenous lives [18]. The research was led by a Torres Strait Islander man who interviewed participants one-on-one using a semi-structured interview guide.

### Data collection

The study was approved by The University of Adelaide Human Research Ethics Committee (H-2015-008) and the Human Research Ethics Committee of the Aboriginal Health Council of South Australia (04–15-603). Participants were invited to participate opportunistically until data saturation was reached. To be included in the study, participants had to be male, over 18 years of age, and of Aboriginal and/or Torres Strait Islander descent and reside in either South Australia (SA) or in Far North Queensland (FNQ), and with the ability to provide informed consent. The sample sites were chosen due to convenience and the networks and relationships that the researcher had in these places. Participants were offered a $50 gift voucher as a token of appreciation for their time and input.

Prior to each interview, the study was explained with the aid of a participant information sheet. It was estimated that interviews would take no more than 1 h to complete. Informed consent was obtained to audio record and transcribe the interview and analyze the data for the study. Interview recordings were transcribed verbatim for analysis using a professional transcription service. Participant’s names were removed from transcriptions as to preserve the anonymity of the interviewed participants.

The semi-structure interviews comprised of broad, open-ended questions. Questions focused on their experiences of health service use, including as a child. Questions for utilization as a child included who took you to the doctors and where did you go. As adult’s participants were asked about motivators of primary health service use, where do they go, what services do they use, and why do they go there? Participants were also asked if they have had any positive or negative experiences using PHCSs and if they had any suggestions or ideas on how services could be more appropriate, inviting, or better engage Aboriginal and Torres Strait Islander men.

### Data analysis

A thematic analysis was applied to the data collected by the semi-structured interviews. Thematic network analysis was chosen because it can “systematize the extraction” of themes into three distinct categories, the lowest level or basic themes, which are grouped together to form organizing themes that have higher order super-ordinate themes known as global themes [3]. Such a technique allows the data collected to be represented as a web-like map illustrating the relationship between the three levels [3]. This process was completed manually without the use of computer software. KC and SH coded the data from the interviews independently before coming together to discuss and negotiate the identified themes. A sub-group analysis was conducted to determine if the findings from men who were employed as a health professional had different experiences to other participants, however, this was not found to be the case and has not been reported here.

## Results

**Fig. 1:**
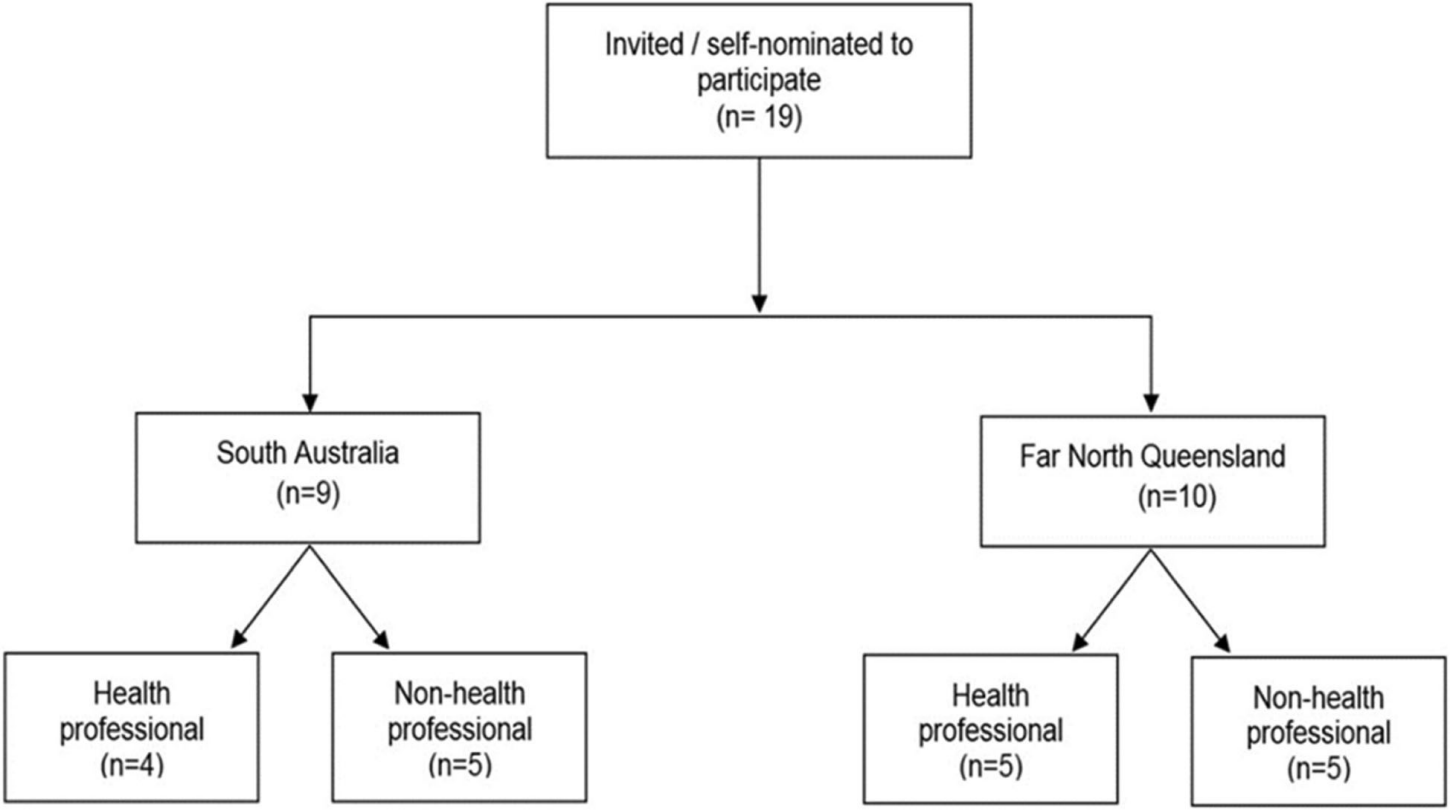
Flow diagram illustrating participants setting and highlighting those employed in the health and or health research sector

Participants were recruited from SA (*n* = 9), and FNQ (*n* = 10). Eligible men were either invited to participate in the study or self-nominated and approached KC as they knew someone else who had participated. Although participants were eligible from all settings, all those interviewed in SA resided in an urban setting. Of those from FNQ, 8 resided in an urban setting with an additional 2 from a remote setting. Participants’ ages ranged from 19 to 65 years. Of those interviewed, 9 were employed as health professionals (working for a health service or health research institute), four from SA and five from FNQ (Fig. 1). Interview lengths were an average of approximately 17 min, with the longest being 30 min in duration.

### Childhood experiences

As children, most men reported that their mothers took them to the doctors; 63% (*n* = 12) reported that it was only their mothers, 16% (*n* = 3) said that their fathers also sometimes took them and 21% (*n* = 4) of participants had other carers such as grandparents, an aunty or their nanny take them to the doctors in addition to or instead of their mother. The common themes regarding why it was the person they had identified who took them to the doctors as a child and not someone else, were availability, perceived role, having relevant health knowledge and rapport with the doctor or clinic. Both the availability and the perceived role of the carer were the primary reasons given.

Yeah, Mum did all that [take kids to the doctors] …Yeah, obviously Dad’s working and – I guess it was more comforting to go anywhere with mum, and especially if you’re sick you would rather be with Mum than Dad, yeah, well that’s how I felt. (Participant K).

Researcher: when you were younger, who was the person who took you to the doctors or health service …?

Participant P: My mother and my grandparents.

Researcher: And why was it your mum and your grandparents? Do you know why?

Participant P: Because they were more like my primary carers, and they were available all the time, to do it.

Carers who didn’t take the participants to the doctors were not available due to other commitments. “He [Dad] worked a lot…so it made it impossible sometimes for him to take us” (Participant D), whilst some men thought their father didn’t believe it was his role, “It is a bit funny that Dad never took me. Maybe he just thought it wasn’t his job…role in the family.” (Participant G)

For others the fact that their carer had relevant health knowledge was a factor, “Mum, being a nurse herself…it was probably more to do with her knowledge” (Participant C), or a rapport with the doctors or clinic, “Mum worked at the doctors, so it made it easier, so we had a close relationship with the doctor that she worked at.” (Participant D)

The reasons participants recalled being taken to the doctors as a child was not asked by the researcher, however, some participants mentioned going to the doctors as a child for illnesses/sickness (*n* = 3), injuries (*n* = 1), prescriptions (*n* = 1), or immunizations (n = 1). No one mentioned (as a child) going to the doctors or PHCSs for check-up or for any other service at the primary health care clinic other than seeing the doctor.

As a child all participants attended non-Indigenous health care clinics or private general practices. Only one participant also remembered attending an Aboriginal Health Service as a child, although it was not their regular clinic. “I’m sure there were occasions of going to an Aboriginal Health Service, but I don’t recall that as much as I do going there [Private GP] visiting the family doctor.” (Participant A).

For some participants attending an Aboriginal Health Service was not an option. For example, two participants interviewed from remote Far North Queensland live in communities that do not have an Aboriginal health service. In addition, one participant who grew up in regional South Australia also expressed that Aboriginal health services were not available. “There wasn’t an Aboriginal specific health service around, growing up in [home town].” (Participant F).

### Adult experiences

The experiences of participants as adults was analyzed and the factors that influence service utilization were classified into three main subthemes; motivators, barriers and enablers. These have been briefly listed in Table 1.

**Table 1:** Factors influencing utilization of primary health care services by Aboriginal and Torres Strait Islander men

Motivators	Barriers	Enablers
• feeling sick/unwell. • for a particular service (Dental or sexual health). • check-ups and preventative health. • family encouragement.	• feeling invincible. • feeling shame or uncomfortable about attending clinic and/or talking about health issues. • fear of getting bad news. • waiting times to get appointments and appointment not running on time. • culturally inappropriate service and undertrained staff. • limited knowledge of other options. • limited availability of other options.	• convenience (opening hours/ proximity to home or work). • the perceived quality of the service. • feeling culturally safe with health service and/or staff and/or having a sense of belonging. • rapport with health staff and/or services.

As adults, participants reported there were four main motivators for going to their doctors or PHCSs; feeling sick/unwell, for a specific service (dental or sexual health), or for check-ups and preventative health to better look after yourself and family encouragement.

There were a group of men who only went when they had to (when they were acutely unwell), for example, “Researcher: And what about now, do you ever visit a health service or a doctor? Participant E: No. I’d need to be dying to do that, brother [laughs].”

There were also others who would only go for a specific service, “I’ve only been to the Aboriginal services for dental stuff” (Participant D). Whereas other men are more proactive and attend for preventative health and have regular check-ups, “I try to get around to get regular health checks” (Participant N), “I visit [the Aboriginal Health Service] and get a full health check every quarter, full bloods, everything…I’m one of those rare blokes that goes out and looks after his health” (Participant F).

Many of the men talked about caring for their health for the sake of their families, “that’s a big thing that men want to be around for their kids…so even if you are busy you should just take the time [to see the doctor]” (Participant J). Others reflected on the experience of their fathers and how that has motivated them to be more proactive regarding preventative health care:

One of the key factors [reasons for going to the Doctors] that I’d suggest is … for your family’s sake, go and get checked so that they can have you around for longer, … my Dad, he passed away from a heart attack, and I’m pretty sure, like if he was – I don’t know, … more inclined to go to the doctors, he might have been able to change his dietary habits, or a few other things in his normal lifestyle activities to be able to prevent that, and yeah, he might have – he might still be here today (Participant C).

Another motivating factor is being encouraged by family members to go to the doctors. “…I go when the wife tells me to” (Participant J), and as another participant explained;

I only sort of took it seriously in the last few years, and that’s because my partner keeps pushing me to do that, and obviously, her being a medical person herself, she has encouraged me to go and get that stuff checked, whether it’s your well health check, go and see a dentist, all this sort of stuff (Participant G).

The reasons participants reported avoiding the doctors fell into five main categories; feelings of invincibility, shame, uncomfortableness and fear, and extended waiting times.

Some men felt there was a general mentality of invincibility “…another reason is just being a male, I suppose. You think you’re 10-foot-tall and made of bricks and nothing is going to go wrong” (Participant G). Feeling shame was identified as a barrier “Sometimes you feel shame to go in and describe your medical condition…most men may be shy to talk about their medical issue” (Participant A), as was feeling uncomfortable at primary health care centers or doctor’s clinics;

I mean in the past when I went into non-Indigenous services …you go inside and you sit down and you feel like – it’s a feeling of feeling uncomfortable because people are looking at you and then just – not even as an Indigenous male, also as a male I – I go to … try and move away from people looking at me and go pick up a magazine and it’s Woman’s Weekly, it’s New Idea, so then things like that too, you know, so it makes – it makes not only – I don’t think Indigenous men, non-Indigenous men too could make a feeling of being uncomfortable (Participant N).

PHCSs and hospitals were often feared. Fear of getting bad news, like you had a medical problem, or having a bad experience whilst in care:

I think, it’s more to do with that, kind of, a little bit of fear, so you don’t know what’s going to happen when you get there, it could be a whole bunch of stuff that he says that you’ve got (laughs), and you are worried that something big might happen, so that plays into it, but also really because the atmosphere is not that flash (Participant E).

Waiting times to get an appointment were described as another barrier. “I think the problem with any good doctors is being about to get bookings…I think availability of a good doctor is sometimes limited” (Participant J). As were the time you can be left waiting at the clinic for your appointment,

You go there, you sit there, and you wait, and you wait, and you wait, and they’re never on time, and it could be 45 min waiting to see somebody, and that’s problematic, it’s a pain in the butt, I’m a busy person (Participant E).

As adults, participants (*n* = 12) were more likely to attend Aboriginal Health Services. Common themes for attending the clinics they choose as adults were; convenience, the perceived quality of the service, feeling culturally safe, rapport and a limited knowledge or availability of other options.

Participants who choose non-Indigenous services tended to report choosing the service because it was convenient. “…it’s just around the corner” (Participant D). Many participants had a very practical approach to service utilization. “No matter where I’m sick I just go to any medical center that’s open at the time” (Participant R). “I will go to a doctors’ [GP service] …access the primary health care center [Aboriginal health service] or a 24-hour service…I think for myself a 24-hour service is probably … it’s easier for me to access” (Participant O).

Participants also often mentioned that the Aboriginal service was not conveniently located. “…it’s not always convenient for me to go there [Aboriginal Health Service] because I lived in the suburbs” (Participant G).

Participants who chose to attend an Aboriginal Health Service reported choosing this service for very different reasons. The reasons described were feeling culturally safe, “We’re comfortable around our own people and that’s just the way it is …It’s just when you’re with black people, you’re different, and you’re understood. You don’t have to explain yourself. It’s just the way it is” (Participant A). “They’ve been around for a long time, they understand the cultural protocols around that kind of stuff and they try to accommodate and cater for your needs” (Participant P).

There was also a sense that the quality of the service received from an Aboriginal health service was superior to that of a non-Indigenous service due to the focus on holistic health:

I know that the GP’s only going to ask me about what I’m there for at the time, so why am I here? Why am I sick? Whereas if I go to the Aboriginal Health Service then I know the doctor will ask more about other things rather than just my presenting illness or symptoms. (Participant A)

Well they [Aboriginal Health Service] always take the time just to talk to you about how you’re feeling, talk about any previous health issues that you’ve had … and you’re talking - in the clinic room for 45 min maybe half an hour, as opposed to three minutes and you always feel a lot happier walking away from there, knowing that you’ve got exactly what you went there for and there’s that bit of connection and relationship that you don’t get to build with the mainstream GP. (Participant H)

Although some participants felt you would get the same service no matter where you went, “I mean, at the end of the day, they’re all qualified people, and they, I guess, all got the same sort of education, and know what they’re talking about… I just went to wherever was accessible for me” (Participant G).

For others, rapport or familiarity was important when choosing a service. This was common for both participants who choose an Aboriginal Health Service and those who attended non-Indigenous clinics or general practitioners. Participants tended to stick to one doctor. “It’s [non-Indigenous GP clinic] the only one I’ve been to and so – well he knows me, so that’s the one I feel comfortable going to about stuff” (Participant D). “He [GP at non-Indigenous clinic] knows me and he’s always good always tells you the truth” (Participant M). Even if they did not have ‘a’ GP, participants were more likely to go somewhere where they felt they had built rapport with the service. “I know some of the people there [Aboriginal health service] so there’s a good GP there for me” (Participant Q).

Other reasons for choosing a non-Indigenous service over an Aboriginal one was because they were not even aware that there was an Aboriginal Health Services available:

Researcher: Do you ever go to the health service here? Because there’s an Aboriginal health service here in the city, do you ever utilize that?

Participant B: No, no.

Researcher: Why not?

Participant B: I don’t know. I’ve just never really heard about it.

Or they did not know where they were located. “I’ve never sought out an Indigenous health center or anything like that, mostly because I don’t know where they are” (Participant K).

### Negative experiences

Some participants shared stories of negative experiences with PHCSs. The themes from these experiences were personality clashes with staff, long waiting times in clinics, culturally inappropriate services and undertrained staff. These experiences were described as reducing their likeliness to return to the service, “I’ve had some bad experience with – just personality clash with a nurse, or staff and doctors here, which has turned me off attending after that” (Participant K), whilst others vowed to never return:

Participant G: I think it was at [Aboriginal Health Service] and it was probably when I first came to [location withheld], so I was only a young person then. I can’t even remember why I went there, but there was a non-Indigenous person that was my first point of contact at reception, and they, basically, just said, ‘Just take a seat, I’ll be with you now – in a sec,’ and I was sitting there for probably an hour, and it was just - like I said, I was a young Aboriginal person. I was only 17 or 18…and that was my first experience, I guess, with the Aboriginal health service, with a non-Aboriginal person at the front desk and told me to sit down, and wait, and they would be with me in a second, and I was there an hour later, and I did – I actually just walked out.

Researcher: And how did it make you feel?

Participant G: Probably not worthy, sort of thing, like, yeah, like, I was a bit pissed off to tell you the truth. I walked out and was, like, well, I’m not coming back here again, and I don’t think I ever did go back there.

This was also expressed by another participant who spoke to the researcher about health services in a hypothetical situation. The participant raised issues of cultural inappropriateness with under-trained health service staff, the absence of cultural protocol, the need for patients to be asked if they have a preference for their care givers gender and the potential impact these issues can have:

Participant F: Well, let’s use a hypothetical. I’m a countryman, been through law,^1^ I come in, I’m expecting in my mind that I’m seeing a male doctor, Aboriginal or not. I go in, go into a small room, I’m waiting, it’s very clinical even in this Aboriginal Health Service, it’s a very clinical examination room, and a white woman walks in. I am going to clam up, I’m not going to say anything and I’m going to put my health at risk, because I’m not going to talk about why I’m there, I’ll make it up and walk out. And at the end of the day I’m there for a service, and I should have been told who I was going to see or had the opportunity to voice my request about saying, I want to see, one, an Aboriginal doctor, an Aboriginal male doctor or a male doctor, those choices need to be there. Of course, hypothetically and in a perfect world, that is the scenario. But when you’re talking about what risks are going to take place from a countryman coming down, that is a massive risk. These guys are traditional men, they want to work in traditional ways, they want to speak with another male, and, of course, this is all hypothetical, I’m making a lot of generic assumptions here [pause] but what we’re trying to do is highlight risks, and those risks are that I may not proceed with an examination and put my health at risk. And we’re talking about physical health, what if it’s mental health, there’s a whole another can of worms. If we’re talking about mental health and I’m going there for a mental health examination or part of a mental health plan, I could become very upset, I may become violent, I may take or not take the correct medication. So, again, there’s a massive ripple effect that can come from one consultation when they get it wrong.

### Suggestions to improve service accessibility, appropriateness

Participants were forthcoming with their suggestions for improving primary health care service utilization by Aboriginal and Torres Strait Islander men. The suggestions varied a lot and were classified into four organizing themes; accessibility, promotion of services and communication between services and clients, cultural appropriateness and gender specific services.

Some suggestions for improving service accessibility were; increasing opening hours, bringing services to men or giving them paid time to see the doctors, making services free of charge and providing incentives for check-ups.

Many men work full-time and attending PHCSs during normal opening hours can be difficult. “You’ve got to give people the option, whether it be between nine-to-five or after-hours or on a weekend” (Participant A). Flexible services that can come to workplaces or workplaces offering men paid time to see the doctors could also help increase utilization. “And I say the doctor should go to the workplace, maybe the workplace should actually incorporate a day off. Where you can have a day off where you go to the health care center maybe, that you’re given a day off paid leave, but you have to go and have a doctor’s check-up” (Participant J). Additionally, free health care services were another suggestion to increase service utilization.

“Researcher: And how can the doctor’s or health service better suit your needs? Participant B: Make it free”. To encourage Aboriginal and Torres Strait Islander men to have check-ups incentives were suggested. “Incentives, that’s what they need, they need incentives, yeah, anything like that” (Participant Q).

The participants thought the promotion of services and improved communication between services and clients could increase engagement between PHCSs, themselves and other Aboriginal and Torres Strait Islander men. “Maybe some social media because everyone is doing that sort of thing. So maybe, just get some advertising through there” (Participant B). Participants also thought that it would be worth **s**ending out reminders to clients that they are due for a check-up. “They [GP or health service] could send notifications to you to say you haven’t had a check-up… or something like that” (Participant J). Participants also felt they were not knowledgeable on when they should be going to the doctors for check-ups and thought that promoting at community events or directly to clients would encourage some men to attend. “…better promoting of what you need to go and get checked up on, like, what year, how old you are. When you hit a certain age, you’ve got to go and check this or check something else out” (Participant M).

Participants thought that some services could do more to make their services and their staff more culturally appropriate. Some of themes included the use of language by staff, the inclusion of interpreters, training for staff and generally making clinics friendlier (less clinical/sterile).

Participants suggested that PHCSs staff could be more culturally appropriate by modifying their language when talking to clients:

Well I think the doctors and nurses have got to be more, I guess, I don’t know the words, but be more casual and be more forthcoming, you know, and talk…just the personal greeting is, you know, they could be out there, be more open, be more like how we interact with each other, openness, a bit loud, and, yeah, I guess - and the greetings in maybe, you know, a couple of the local languages is always good (Participant K).

One participant explained his perfect health center would be inclusive, accessible, and casual and have interpreters for clients:

Researcher: If you could design your own health service, like, you’ve got all the bucks in the world, what would your health service look like and who would you employ…

Participant I: Yeah. I would set-up a good health center for everyone, not only us as the Aboriginal and Torres Strait Islander people, but for everyone to come who cannot access doctors or clinics, like, 30 km down the track, you have to drive an hour there, an hour back, something that’s closer to everyone, and also that there’s a place that they can hang around and have barbeque once a month [laughs] or when they come that they have something to eat, and they see the doctor after lunch, something like that, yeah.

Researcher: What about staff?

Participant I: Yeah. I would employ Aboriginal and Torres Strait Islander people, but also you’ve got other communities here, and you get that one can interpret because we all speak different language, you’ve got all the multicultural here, so you get one person, as a health worker, can talk in their language to their community or whoever come to visit the health clinic, so they can sit with the doctor and explain and interpret what the doctor say and they know that, because many of our old people don’t speak much English, and also the people now coming to Australia, all those refugees, so it’s good to have an interpreter working there.

One participant shared his experience as a health professional and how the Aboriginal health service he works for values getting education about the importance of health checks out there to the men in a culturally appropriate way during men’s group camps:

…on the camp, we have this camp, and we sit around, we yarn, we yarn about men’s issues, we yarn about men’s health and everything like that. And, then, they tell us – tell me what’s wrong, some of them do, some of them open up. Some of them just keep it to themselves, but they’re listening, yeah. And that’s about getting their message out, that, don’t be frightened of the health check. That’s all we can do, just tell them, just give them an education – why. Because our men die at a young age, now (Participant Q).

In relation to building or modifying PHCSs to reduce barriers and improve utilization, the heterogeneity of Aboriginal and Torres Strait Islander men was acknowledged by some participants, and that a one-size fits all approach will not work.

Participant F: We need to target and hone in on the right cohort. If we’re focusing in on Aboriginal men we have to use language suitable to Aboriginal men, and that means changing the language from region to region and the way that we focus on the men would be different from region to region. Let’s not fall in the mistake of putting Aboriginal and Torres Strait Islander men under one blanket that it’s one culture, because we know it’s not. So, it has to be very [location] specific.

Participants were asked if they thought gender specific health services would improve the utilization of PHCSs by Aboriginal and Torres Strait Islander men. Most of the participants (*n* = 15) agreed it would be a great idea and something that they would support, “I think they need to have men only days – where with men only days, men only staff, men only – like on a weekend…” (Participant A).

Participants thought seeing male doctors was more appropriate, “I think talking to men is probably more healthier than me talking to a woman about my problems or my issue, or it’s easier” (Participant L), and that they would feel more comfortable than they would with a female doctor, “Obviously, I’d love – I’d always like to see a male. I feel more comfortable speaking to a male, especially if I had male problems. I’d rather speak to a male than a female” (Participant D). Even though five participants said that they didn’t mind if they saw a male or female doctor, if it was particularly about sexual health issues, 9/13 participants would prefer to see a male doctor, some expressing that it would be completely inappropriate to discuss these topics with a female doctor and they would refuse to do so. “If we’re talking about man’s things, I’d be more comfortable talking to a male doctor. If they [doctor] are female I wouldn’t go, I’d walk out” (Participant K). A few men, 3/13 said the gender of the doctor would not bother them and 1/13 said that they would prefer a female doctor to discuss sexual health issues.

## Discussion

Our study found some men to be motivated to attend PHCSs for check-ups and/or for preventative health, and given more favorable conditions, more of our participants would be motivated to do the same. This finding is similar to results from Smith et al.’s research which found men in their study “were willing to speak about their health in an open manner when provided with an appropriate environment in which to do so” [20]. Although some of the men in our study acknowledged going to the doctors only when they are “dying”, data from this study challenges the stereotype that men are disinterested in their health and avoid going to the doctors.

This study found some of the factors that influence the participant’s decision to access/choose a primary health care service to attend were; convenience, the perceived quality of the service, feeling culturally safe and rapport with the health staff and service. Similar findings were also highlighted in studies by [1, 13, 15, 16].

When a participant is acutely unwell, convenience is a major deciding factor and participants may choose a service based on its location or opening hours regardless of perceived quality of the services provided. Providing access to high quality health care service access for Aboriginal and Torres Strait Islander people is a program objective of the Indigenous Australians’ Health Programme [4]. Convenience was also mentioned by Hayman who highlights how Indigenous patients used their service because it was convenient as they lived nearby [12].

Cultural safety and culturally appropriate staff and services are important factors as found in our study which mirrors findings from the studies by [1, 13, 15, 16]. Culturally safe spaces and appropriate staff can be a powerful enabler. Equally, feeling unsafe or feeling that a service or staff are culturally inappropriate is an important barrier. More can be done to make PHCSs culturally appropriate, examples of factors that foster cultural safety found in our study and the review by Isaacs et al. includes cross-cultural/cultural competency training, employing Indigenous staff and gender specific staff and services [14].

Some participants in our study said they avoided the doctors because of feelings of invincibility. This was like findings from qualitative studies on the utilization of sexual health services [1] and mental health services [15, 16] by Aboriginal and Torres Strait Islander men, as did a literature review by [14] on the barriers and enablers to utilization of adult mental health services by Australia’s Indigenous people. Similarly, Isaac’s studies [15, 16] found that men expressed the need to be ‘strong’ as a barrier for accessing mental health care.

Our study also found some men are not disclosing or discussing all their health concerns due to feeling uncomfortable or shameful about their condition, feeling culturally unsafe, or fearing the unknown or that they will receive bad news. This has been found in previous studies [1, 15, 16], a review of mental health service utilization [14] and discussed in commentaries by respected Aboriginal and/or Torres Strait Islander health leaders, Dr. Mark Wenitong [21], Dr. Noel Hayman [11] and Mr. Aaron Briscoe [5]. Adams et al. [1, 15, 16] found that stigma around sensitive health issues (sexual health and mental health) was a barrier to service utilization. Culturally inappropriate services and/or staff was another identified barrier that was also found in previous studies [1, 15, 16], discussed in a review [14] and commentary piece on engaging Indigenous men [21].

Participants in this study also wanted PHCSs to make it easier to get appointments and have clinics run on time to reduce waiting periods would reduce barriers. Similarly, Hayman explains how clients long waiting times were “reasons for not attending” the health care service [12]. Participants also advocated for out-of-hours services which Hughes’s study also found when men wanted an “a 24-hour doctor phoneline” [13]. Bringing services to men (outside of the clinic) and having services free of charge are two findings highlighted by [12], who has been asked to consult many male patients at home, and by Hughes who states that cost of care were issues when accessing health services [13]. Wenitong et al. also believes that lack of transport can be a limiting factor, therefore providing transport may increase access [21].

Participants in this study felt that PHCSs could also do more to promote their services so that men know where they are and what services they provide. Some participants suggested services send reminders to patients, so they know when they should be attending check-ups. Danila Dilba men’s only health service in the Northern Territory provides clients with a “courtesy reminder about their appointment the day prior” [8].

Participants in our study also felt that they needed to have access to more information about when to go the doctors for check-ups. This was also echoed by [1] and Isaac’s studies [15, 16] where participants thought they need more information on recognizing signs and symptoms. However, unlike the previous studies, this study did not find concerns about confidentiality or distrust of the health services as barriers [1, 15, 16].

Many men in our study supported the idea of gender specific services and/or times when a clinic is opened for men only and is staffed by men. Hayman [11, 21] also advocate for men’s only clinics as strategies for reducing barriers for Aboriginal and Torres Strait Islander men to access PHCSs. Danila Dilba’s Health Service Men’s Clinic, Northern Territory, has been successful in engaging their intended clients by offering a comprehensive service staffed by a practice manager, clinic coordinator, Aboriginal health practitioner, two general practitioners, a customer service officer and a counsellor; all of which are men [8]. Danila Dilba also holds “specialist clinics with a visiting Endocrinologist every 3 months” [8].

A few studies have explored how Aboriginal and Torres Strait Islander men utilize PHCSs in relation to specific health services; reproductive health [1], mental health [15, 16] and one study on the health seeking behaviors of Indigenous men from Hawaii [13]. There has also been some published expert commentary on the subject [5, 12, 21], one of which was specific to engaging Aboriginal and Torres Strait Islander men about sexual health issues [21]. This study is however the first, to our knowledge, that has explored the engagement of Aboriginal and Torres Strait Islander men with PHCSs without narrowing the focus to a specific health topic.

This small qualitative research study had many strengths and some limitations. One of the key strengths is the study followed the principles of Indigenist research; the research has arisen from the long history of oppression, looks to move forward from colonization to support the struggle of Indigenous people to heal, the study was designed by and interviews were facilitated by an Indigenous man, and the data analysis privileged the voices of the Aboriginal and Torres Strait Islander men [18].

The results may not be representative of all Aboriginal and Torres Strait Islander men across Australia as it was a small sample. There is a potential selection bias as recruitment was opportunistic, however, the researchers aimed to get a wide selection of participants. Participants were of both Aboriginal and/or Torres Strait Islander decent, differing ages, backgrounds, careers and health status. Interviews were conducted with men from urban South Australia and in both urban and remote Far North Queensland giving as broad a sample as could be obtained within the project’s capacity.

The study participation rate was 100% as none of the men invited to participate declined the opportunity and in addition, some self-nominated and approached KC to be involved in the study. This may be due to the established rapport that the lead researcher (and interviewer) had with participants through relationships or association, and/or reflect the interest of participants being asked their input and opinion on the important issues of assessing PHCSs. To minimize potential bias in response, most of the interview questions were open-ended.

## Conclusion

Aboriginal and Torres Strait Islander men have the highest rates of morbidity and mortality in Australia yet are poor utilizers of PHCSs [5]. Informed by 19 interviews with Aboriginal and Torres Strait Islander men from South Australia and far north Queensland this study found that men do seek preventative health care services, the use of which is influenced by multiple interrelated motivating and enabling factors and barriers.

This study was able to show that Aboriginal and Torres Strait Islander men are interested in their health, and when given the opportunity, are willing to share their ideas and suggestions as to how service utilization could be improved. This study also revealed how the development of local strategies should be co-founded by local Aboriginal and Torres Strait Islander men together with their local PHCSs. This is a good starting point for self-reflection and should be used as a guide towards informing strategies and initiatives that could increase utilization.

This study uncovered the need for future studies about the effectiveness of gender specific PHCSs, how childhood experiences effect utilization as adults, and the importance of better understanding the difference between those who are motivated to attend PHCSs for preventative health and those who are not. All of which have the potential to increase primary health care service utilization. Finally, acknowledging the heterogeneity of Aboriginal and Torres Strait Islander men, communities and PHCSs will assist key stakeholders in avoiding wasting valuable resources on a one size fits all approach.

## Abbreviation

PHCSs: Primary health care services

## Footnotes

1 To be initiated, one’s right to passage. Occurs when adolescent boys or girls have proven themselves to be mentally and physically worthy of adulthood.
